# Splenic Flexure Volvulus Managed With Left Hemicolectomy and Primary Anastomosis: A Case Report

**DOI:** 10.7759/cureus.78579

**Published:** 2025-02-05

**Authors:** Blanche Lee, Bianca Kwan, Nikhil Narsey

**Affiliations:** 1 General Surgery, Toowoomba Hospital, QLD, AUS

**Keywords:** acute large bowel obstruction, acute volvulus, emergency laparotomy, splenic flexure volvulus, surgical emergencies

## Abstract

Volvulus of the splenic flexure is a rare cause of large bowel obstruction. We present the case of a male in his mid-20s who presented with a three-day history of colicky left-sided abdominal pain. A computed tomography scan revealed severely dilated large bowel loops with a closed-loop obstruction in the left upper quadrant, which was thought to originate from the transverse colon. An urgent laparoscopy was performed, and a splenic flexure volvulus was found with a 720-degree rotation around its mesentery. The bowel was viable with no evidence of ischemia or perforation. There was a congenital absence of the splenic flexure ligamentous attachments. It was not possible to safely devolve the volvulus laparoscopically, and the operation was converted to a laparotomy. A left hemicolectomy with a primary anastomosis was performed to remove the pathologically dilated and mobile portion of the bowel. The patient made a good recovery.

## Introduction

A volvulus is the abnormal torsion of the intestinal tract around its mesenteric attachment and is the most common nonmalignant cause of large bowel obstruction (LBO). While volvulus commonly occurs in the sigmoid colon and cecum, splenic flexure volvulus is rare and accounts for approximately 2% of cases [1]. Features that may predispose to a volvulus include a redundant colon with a narrow mesenteric attachment, non-retroperitonealized structures, and colonic dysmotility. The splenic flexure is typically stabilized by peritoneal attachments, which makes volvulus in this location rare. A volvulus may represent a surgical emergency if there is an obstruction or impaired vascular perfusion due to the risk of bowel ischemia, gangrene, and perforation.

## Case presentation

A male in his mid-20s was referred from a primary care center to the emergency department with a three-day history of colicky left-sided abdominal pain and anorexia. He had passed flatus the morning of his presentation and had one episode of vomiting three days prior. He had no previous medical or surgical history and reported a normal bowel habit before presentation. There was no family history of connective tissue disorders. His vital signs were normal. On examination, his abdomen was soft with tenderness in the left upper quadrant. There were no signs of peritonism. Blood analysis was unremarkable and included normal hemoglobin, white cell count, and lactate. His electrolytes were normal, and renal function was preserved. 

**Figure 1:**
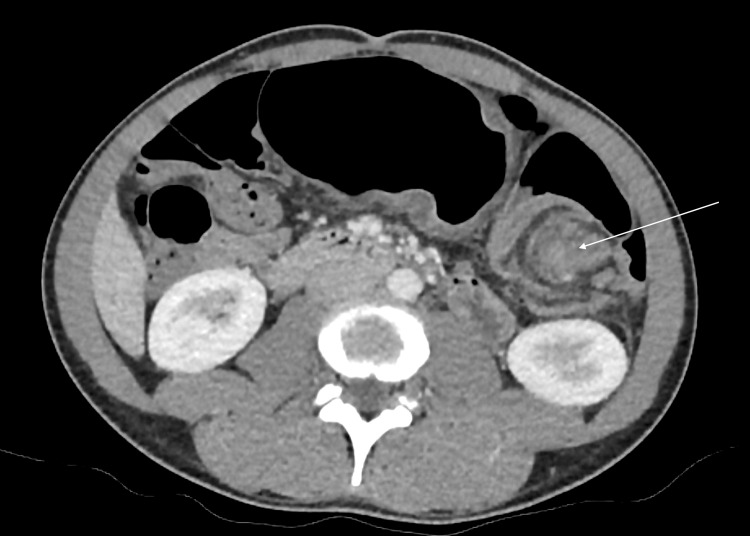
Axial CT view of the abdomen. Dilated large bowel loops with a *whirl sign* of the mesentery (arrow) are seen in the left upper quadrant of the abdomen.

**Figure 2:**
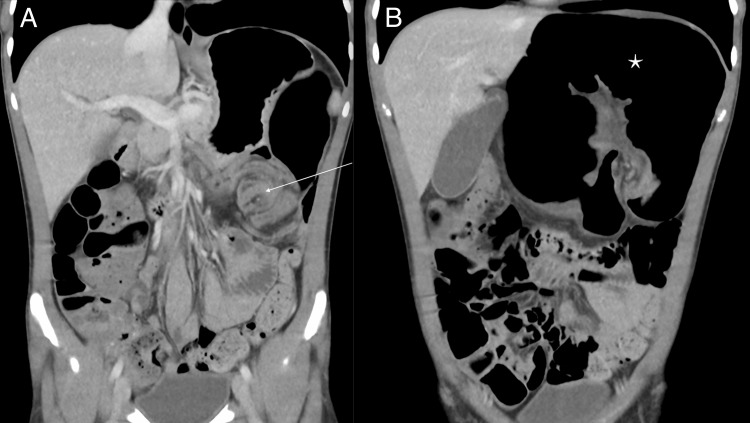
Coronal CT view of the abdomen. Pane A demonstrates dilated large bowel loops with a *whirl sign* of the mesentery (arrow). Pane B illustrates the *coffee bean sign* (star).

A computed tomography (CT) scan of the abdomen revealed severely dilated large bowel loops, raising concern for a closed-loop obstruction in the left upper quadrant. A *whirl sign* of the mesentery and a *coffee bean sign* of a twisted, gas-filled colonic loop was observed in the left upper quadrant (Figures 1, 2). The loop of bowel appeared to originate from the transverse colon.

**Figure 3:**
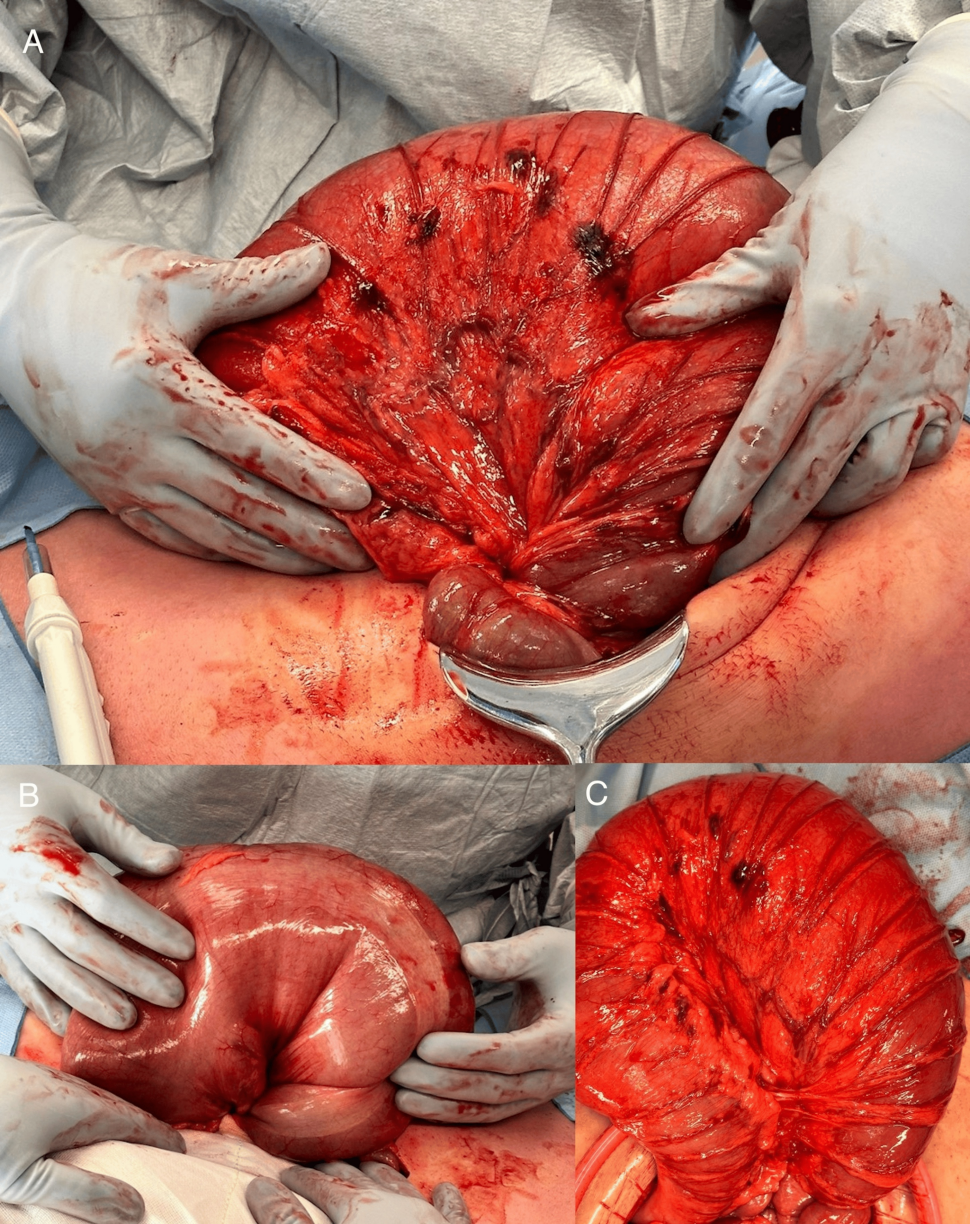
Operative photographs of a splenic flexure volvulus. Pane A depicts the anterior view, and pane B depicts the posterior view of a freely mobile splenic flexure volvulus with a 720-degree twist. Pane C shows the devolved loop of bowel.

The patient was taken to the operating theater for an urgent diagnostic laparoscopy. A splenic flexure volvulus was found with a 720-degree rotation of the bowel around its mesentery. The bowel was severely dilated but viable. There was no evidence of ischemia or perforation. The proximal transverse colon and mid-descending colon were of normal caliber. It was not possible to safely devolve the volvulus laparoscopically, and the operation was converted to a laparotomy. The volvulus was delivered and devolved (Figure 3). There was a congenital absence of all splenic flexure ligamentous attachments.

A left hemicolectomy was performed to remove the pathologically dilated and mobile portion of the bowel. An isoperistaltic colo-colonic anastomosis was created between the mid-transverse colon and the descending colon. A drain was left in the pelvis. The patient’s postoperative course was complicated by urinary retention and ileus. The patient was initially placed on a clear fluid diet with a gradual upgrade to a full diet. A subsequent trial of voiding was successful. The patient opened his bowels, and he was discharged eight days following his operation. 

Histopathology demonstrated markedly dilated large bowel mucosa up to 12 cm. While areas of the bowel wall appeared attenuated, there was no signs of ulceration, perforation, or ischemia. Microscopic sections demonstrated loss of normal mucosal folds with mild architectural distortion. Two lymph nodes were sampled with no features of malignancy.

## Discussion

LBO is caused by a volvulus in 15%-20% of cases [2]. The sigmoid colon and cecum are the most common sites of volvulus, while volvulus of the splenic flexure is rare due to its relative immobility. The splenic flexure is normally fixed in place by three ligaments: the gastrocolic, phrenocolic, and splenocolic ligaments. Risk factors for splenic flexure volvulus include congenital absence of ligaments, chronic constipation resulting in an elongated mesentery, malrotation, iatrogenic disruption of ligaments from previous surgery, and adhesions [3]. Two-thirds of cases of splenic flexure volvulus have been reported as secondary to mobilization of the splenic flexure due to previous surgery [4]. Other risk factors relate to gastrointestinal dysmotility and include cerebral palsy, myotonic dystrophy, and Hirschsprung’s disease. Splenic flexure volvulus has also been observed in patients with Chilaiditi syndrome and a wandering spleen [5,6]. 

CT scan has a sensitivity of 95% and specificity of 93% for the detection of LBO [7]. The management of a splenic flexure volvulus depends on the suspected presence or absence of ischemia, necrotic bowel, peritoneal soiling, and hemodynamic stability. Treatment options include endoscopic detorsion, colopexy, or resection of the redundant bowel. In our case, a segmental colectomy with primary anastomosis was performed, as the patient was young and otherwise healthy, due to the high risk of volvulus recurrence. However, a primary anastomosis should be avoided in cases of significant peritoneal soiling or in the presence of gangrenous bowel due to the high risk of an anastomotic leak [4]. The decision to perform a resection with primary anastomosis, with or without a proximal diverting stoma, or resection with an end colostomy should be individualized based on patient factors [8,9]. Colopexy, involving fixation of the splenic flexure to surrounding structures, is a less invasive option for elderly or high-risk patients [10].

## Conclusions

Splenic flexure volvulus is a rare and uncommon cause of LBO. A thorough history and clinical examination are required, as normal hematological and serum biochemistry tests do not exclude serious abdominal pathology. CT imaging is highly sensitive and specific for the diagnosis of LBO. Prompt management of this condition is required with endoscopic detorsion, colopexy, or segmental resection to decrease morbidity and mortality due to the risk of colonic ischemia and perforation. In our case, laparoscopic detorsion of the volvulus was unsuccessful, and conversion to a laparotomy was required for surgical resection.
